# Novel protein signatures suggest progression to muscular invasiveness in bladder cancer

**DOI:** 10.1371/journal.pone.0206475

**Published:** 2018-11-12

**Authors:** Magnus Berle, Luiza Ghila, Heidrun Vethe, Adeel Chaudhry, Hilde Garberg, Christian Beisland, Øystein Ariansen Haaland, Eystein Oveland, Ole Johan Halvorsen, Thomas Davidsson, Simona Chera

**Affiliations:** 1 Department of Urology, Haukeland University Hospital, Bergen, Norway; 2 Department of Gastrointestinal Surgery, Haukeland University Hospital, Bergen, Norway; 3 Department of Clinical Medicine, University of Bergen, Bergen, Norway; 4 Department of Clinical Science, University of Bergen, Bergen, Norway; 5 KG Jebsen Center for Diabetes Research, Department of Clinical Science, University of Bergen, Bergen, Norway; 6 Department of Biomedicine, Proteomic Unit, University of Bergen, Bergen, Norway; 7 Department of Global Public Health and Primary Care, University of Bergen, Norway; 8 Department of Pathology, Haukeland University Hospital, Bergen, Norway; 9 Center for Cancer Biomarkers CCBIO, Department of Clinical Medicine, University of Bergen, Bergen, Norway; Centro Nacional de Investigaciones Oncologicas, SPAIN

## Abstract

Patients with bladder cancer need frequent controls over long follow-up time due to high recurrence rate and risk of conversion to muscle invasive cancer with poor prognosis. We identified cancer-related molecular signatures in apparently healthy bladder in patients with subsequent muscular invasiveness during follow-up. Global proteomics of the normal tissue biopsies revealed specific proteome fingerprints in these patients prior to subsequent muscular invasiveness. In these presumed normal samples, we detected modulations of proteins previously associated with different cancer types. This study indicates that analyzing apparently healthy tissue of a cancer-invaded organ may suggest disease progression.

## Introduction

Bladder cancer (BC) is the most common cancer in the urinary tract with an estimated 429,000 new cases and 165,000 deaths annually worldwide [1]. The need for life long surveillance and the treatment of the recurring disease elicits a stronger economic pressure than most other cancer types [2–4]. In principle, there are two main categories of bladder cancer, *Non-Muscle Invasive Bladder Cancer* (NMIBC) and *Muscle Invasive Bladder Cancer* (MIBC). The NMIBC does not metastasize to distant location but has a high recurrence rate in the bladder with a need for frequent controls and long term follow-up. The MIBC is associated with a poor prognosis due to local and distant metastases. From a clinical standpoint, the progression from NMIBC to MIBC is the major determinant for performing a cystectomy [5]. Recent studies have started to identify molecular signatures characterizing these two main categories of bladder cancer [6–8]. A principal challenge in translational research is the tumor heterogeneity, adding to the level of complexity for analyses of tumor markers [6, 7]. Personalized therapy is still a distant option because the clinical heterogeneity of BC is considerable.

There are several molecular mechanisms described for NMIBC and MBIC, supporting the claim that NMIBC and MIBC are driven by largely distinct but overlapping molecular pathways [8–10]. Previous studies have identified that altered expression of members of the Ras-MAPK and PI3K-AKT signaling pathways were involved in NMIBC onset. Mutations in the FGFR3, PI3KCA and HRAS genes were strongly associated with NIMBC recurrence risk. In contrast, the MIBC occurrence is primarily linked to defects in cell cycle regulators, especially Rb1 and TP53. Moreover, both Rb1 and TP53 were shown to be involved in the transition from NIMBC to highly aggressive MIBC [11]. In addition, deregulation of senescence markers such as p16, p21 and p27 has been reported as predictors for NMIBC progression [12–15]. Shariat *et al*. showed a direct correlation between the number of altered genes in patients and the predicted outcome [14]. A comprehensive multi-marker panel allowing the accurate detection of the patients’ genetic profile is a prerequisite for the efficient patient follow-up and disease-free prediction.

Throughput and sensitivity of whole genome transcriptomics exceeds proteomic methods by several orders of magnitude. An increasing number of studies reported discrepancies between the observed transcriptional regulation and the outcome at the protein levels [16–18]. In this study, we defined the molecular signature of bladder cancer by characterizing the overall variation in protein expression between the bladder cancer tissue and the normal bladder tissue of the same patient. Our goal was to create a dynamic and comprehensive molecular taxonomy of the processes involved in bladder cancer development. By performing exploratory proteomics of cold cup biopsy material of primary NMIBC material, we identified protein signatures with predictive value for bladder cancer progression towards MIBC.

## Methods

Eight patients with primary NMIBC, age 55–76 yrs., five male, three female, were included in the study. An overview of the patients is presented in Table 1. Two additional patients were a part of this study as far as the proteomics analyses, but were excluded due to pathology not matching the inclusion criteria. The inclusion criteria were no previous history of bladder cancer, no previous treatment of bladder such as mitomycin, Bacillus Calmette–Guérin (BCG), and diagnosis verified by pathology. Samples were collected after informed consent during surgery at Haukeland University Hospital (Bergen, Norway).

**Table 1:** Participants of the study, with age, gender, pathologic tumor stage [19], histologic grade [20–22] and recurrence status.

Patient	Age	Gender	Tumor stage	Grade WHO 2004	Grade WHO 1973	Recurrence status
1	55	m	pTa	HG	2–3	3 months—cystectomy
2	76	f	pTa	HG	2	
3	85	m	pTa	HG	2	7 months—palliative care
4	74	f	pTa	LG + focal HG	1–2 + focal 2–3	
5	71	f	pT1	HG	3	
6	76	m	pT1	HG	3	
7	76	m	pTa	LG	1	
8	74	m	pTa	HG	3	3 months—cystectomy

During operative treatment, cold cup biopsies were collected from the tumor itself and from macroscopically normal bladder surface on the opposite side of the bladder. The normal tissue was at all times biopsied before the tumor biopsies. The biopsies were fresh frozen in sample tubes on dry ice and transferred in frozen state to an ultrafreezer of -80°C. The samples were processed (as described below) and analyzed by LC-MS/MS in a regular label free proteomics workflow. Internal control was made as an equal mix of all samples and run six times as technical replicates by LC/MS-MS during the analyses.

### Histopathological evaluation

Histopathological findings were initially assessed using the three-tiered WHO classification and grading system from 1973 [22], and the two-tiered WHO/ISUP system (low and high-grade) from 2004 [20, 21]. The biopsies and resection specimens were re-evaluated by a uropathologist (OJH) and graded according to these two systems. The primary biopsies from two patients, originally not graded into the low-high grade scheme, were upon re-examination graded as high-grade with original and final WHO grade 3. The area of highest grade in HE stainings were for each patient chosen for comparison with selected markers in immunofluorescence studies (S1 Fig).

### Chemicals

Trypsin was purchased from Promega (Madison, USA). Urea, dithiothreitol *(DTT*), iodoacetamide, acetonitrile (ACN), formic acid (FA), trifluoroacetic acid (TFA), sodium dodecyl sulfate (SDS), ammonium bicarbonate, water and Tris(hydroxymethyl)aminomethane (tris) were purchased from Sigma-Aldrich (St. Louis, USA). Water and ACN were of HPLC quality.

### Sample preparation

The excised bladder tissue was cut into smaller pieces using a scalpel and added 5 μl lysis buffer (4% SDS, 0.1M DTT, 0.1 M Tris/HCl pH 7.6) per mg tissue. The solution was sonicated utilizing Vibra-Cell (Sonics & Materials, Newtown, USA) for 5 seconds followed by 5 seconds incubation on ice until a homogeneous solution was achieved. Further the lysate was incubated for 3 minutes at 95°C and centrifuged for 5 minutes at 16000g to clarify the lysate. The protein concentration was measured by using the Direct Detect (Merck Millipore, Billerica, USA) spectrometer according to the manufactures instructions. The lysates were digested using the Filter aided sample preparation (FASP) method [23]. 20 μg protein were mixed with 200 μl 8M urea in 0.1M Tris/HCl pH 8.5 in a Eppendorf tube (Eppendorf, Hamburg, Germany) for 3 minutes before transferred into a centrifugal ultrafiltration units with a nominal molecular weight cut off of 30,000 (Cat no. MRCFOR030, Millipore) (Merck Millipore, Billerica, USA) and centrifuged at 14,000g at RT for 15 minutes. The same centrifugation settings were used throughout the protocol unless otherwise stated. Another 200 μl of 8M urea was added to the filter and centrifuged as above. The flow-through was discard and 100 μl 50mM iodoacetamide in 8M urea was added and the filters were incubated in dark for 20 minutes followed by centrifugation. Filters were washed three times with 8M urea followed by two washes with 100 μl 50mM ammonium bicarbonate. The flow-through was discard and 100 μl 50mM ammonium bicarbonate was added to the filters followed by 75 μl trypsin diluted in ammonium bicarbonate (Enzyme/protein ratio 1:50). After incubation at 37°C for 16 hours the filters were transferred to a new collection tube and centrifuged for 10 minutes. For elution of peptides the filters were added three times 40 μl 50 mM ammonium bicarbonate followed by 50 μl 0.5M NaCl with 10 minutes centrifugation in between. The eluate was acidified with FA to a final concentration of 1%.

All samples were desalted using a reverse-phase HLB μElution Plate 30 μm (Waters, Milford, USA). Briefly, the plate was washed once with 80% ACN/0.1% FA followed by two washes with 0.1% FA. After adding the sample the plate was washed three times with 0.1% FA. The bound peptides were eluted twice with 100 μl 80% ACN/0.1% FA. All steps used a centrifugation speed at 200g for 1 minute, except for addition of sample where 150g for 3 minute was used. The samples were lyophilized in a Centrivrap concentrator (LabConco, Kansas City, USA) at 30°C and the peptides dissolved in 1% FA/2%ACN.

### LC-MS/MS analyses

The LC-MS/MS analysis was performed according to laboratory standard operating procedures, as previously published in [24]. A summary of instruments, columns and chromatography settings is included in **S1 File**.

### Ethics

The Regional Committees for Medical and Health Research Ethics of Western Norway approved of this study (2009/1527/REK Vest). All experiments were performed in accordance with relevant guidelines and regulations. Written informed consent was received from participants prior to inclusion in the study.

### Statistics

We adjusted for multiple testing by calculating q-values based on p-values following the approach of Storey & Tibshirani [25], utilizing R statistics software version 3.4.3 [26]. Among q-values less than 0.1 we expect 90% to be true. Significance tests with p-values from IPA data analysis is reported from the software. Other p-values are calculated as a regular 2-sided t-test assuming equal variance in Microsoft Excel.

### Data analysis

The LC-MS/MS raw data was analyzed utilizing Progenesis QI v2.0 (Nonlinear Dynamics, Newcastle upon Tyne, UK) and the proteins identified using Proteome Discoverer v1.4.1. (Thermo Scientific, Waltham, USA). In Progenesis QI runs with an MS1 alignment score >66% was accepted prior to quantification of only unique peptides with charges from+ 2 to +4. An mgf file was generated including MS/MS features with a spectrum rank less than 5, a number of isotopes less than 5, and a value higher than zero for peptide ion and precursor intensities. The fragment ion count was limited to 150 and enabling deisotoping and deconvolution prior to export.

The exported mgf file was searched using Proteome Discoverer using Sequest HT and MS Amanda (version 1.4.4) with the SwissProt *Homo sapiens* database v June2015 (canonical sequence, no isoforms). Trypsin was set as the enzyme, and a maximum of one missed cleavage was allowed. Carbamidomethylation of cysteine was set as fixed and oxidation of methionine as variable modification. The precursor mass tolerance was set to 5 ppm and the fragment mass tolerance to 0.05 Da. The peptide spectrum match validation from all search engines was performed using Percolator, with a strict and relaxed target FDR of 0.01 and 0.05, respectively. The search results were exported as pepXML and imported into Progenesis QI where the identities were assignment to the MS1 features. Progenesis was used to generate a PC bi-plot based on all protein regulations. The protein quantification data was exported from Progensis QI for further statistical analyses.

The hierarchical clustering of proteins with dendograms was generated using Perseus 1.5 [27].

Analysis of gene name, alternative gene name versus protein accession number was manually searched through uniprot (www.uniprot.org). Pathway analyses were made utilizing the Kyoto Encyclopedia of Genes and Genomes–KEGG (www.kegg.jp) [28–30]. The genes identified in KEGG pathways, were manually searched for in the dataset and the corresponding proteins were annotated where a single or similar protein could be identified. KEGG pathways were searched for bladder cancer pathway, as well as cell cycle, MAPK, TGF-beta, VEGF and p53 signaling.

The resulting suggested markers of bladder cancer in late review by Knowles and Hurst [31] and research articles by Chen *et al*. [32], Chen *et al*. [33], and Shimwell *et al*. [34] were searched in our dataset.

The proteomics data were explored utilizing Ingenuity Pathway Analysis (IPA, QIAGEN, Redwood City, USA) as described in Vethe *et al*.[35].

The MS proteomics data have been deposited to the database ProteomeXchange consortium [36] via the PRIDE partner repository [37, 38] with the dataset identifiers: PXD010260.

### Immunofluorescence

Collected tissue samples were fixed, dehydrated, embedded in paraffin, and sectioned at 5 μm with a microtome. Paraffin sections were processed as previously described [39]. In short, dewaxed and rehydrated sections were permeabilized in 0.1% Triton X-100, washed, and blocked in 2% BSA and 0.1% Tween in PBS. For antigen retrieval, samples were incubated in 10mM sodium citrate buffer, pH 6.0 and maintained below boiling for 10 minutes in a microwave before permeabilisation. The primary antibodies used for immunofluorescence were rabbit anti-VEGF (1/250; Abcam ab32152), mouse anti-PCNA (1/500; Abcam ab29). After overnight incubation and washing in PBS, sections were incubated with specific secondary antibodies coupled to either Alexa 594 or Alexa 647 (Molecular Probes). Specimens were mounted in ProLong Gold Antifade (Molecular Probes) and examined with a Leica confocal microscope (SP5).

## Results

### Global proteomics analysis identified outliers with potential predictive value for bladder cancer progression

**Fig 1:**
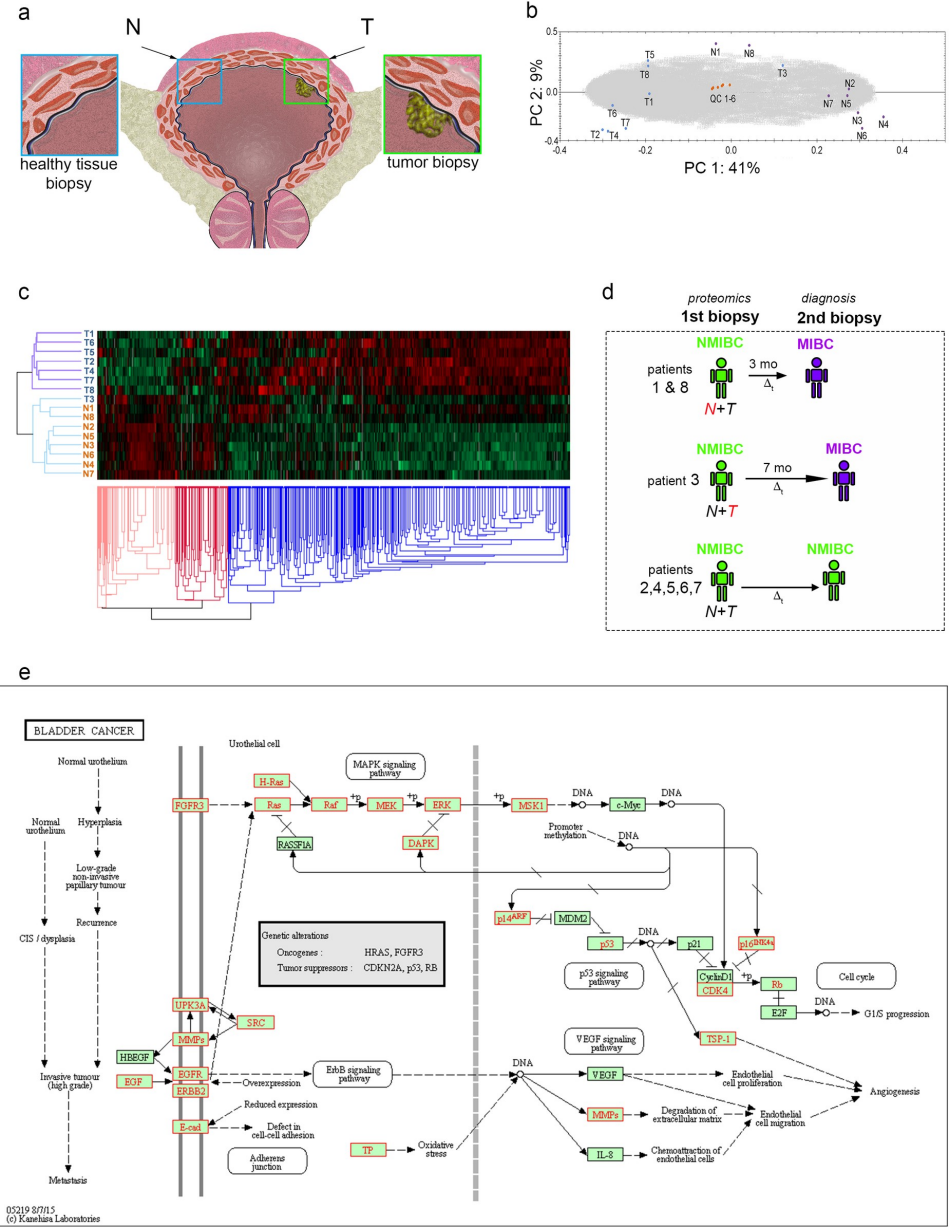
Global proteomics of eight NMIBC patients reveals outliers. a) Cartoon depicting the bladder and the generic location of the biopsies prelevation for proteomics (T = tumor, N = normal). b) PC plot of tumor biopsies “T” and normal bladder “N” from eight patients and six quality control samples. c) Hierarchical clustering with heat map show a separation between tumor biopsies “T”, normal biopsies “N” and illustrate outliers T3, N1 and N8. d) Scheme depicting the time evolution of the outliers (red letters) and typical patients patients (green–NMIBC, purple–MIBC Δt—time interval). e) KEGG pathway of bladder cancer; red marks annotation of proteins identified to genes with known involvement in bladder cancer.

The European Association of Urology and The American Urological Association recommendations define the gold standard approach for staging and adequate diagnosis of BC [40, 41]. For research purposes, we have performed cold-cup biopsies of normal bladder and bladder cancer (**Fig 1A**) in the clinical workflow before transurethral resection.

We identified 4647 proteins, of which 4422 were differentially expressed (DE) between the sample groups (q<0,10). The principal component (PC bi-plot (**Fig 1B**) shows a clear separation of the tumor and control groups with the QC samples closely grouped around zero, hence indicating a high reproducibility in the LC-MS/MS analysis and the label-free quantification. Moreover, hierarchical clustering shows a separation between tumor biopsies “T”, normal biopsies “N” (**Fig 1C**).

Considering **Fig 1B**, we see that the all tumor samples except that for patient 3 (T3) are clustered together along the first principal component, as are all the control samples except those for patients 1 (N1) and 8 (N8). From now on the samples T3, N1 and N8 will be referred to as ‘the outliers’. Patient 1 and 8 were in clinical control identified muscular invasiveness and both underwent a cystectomy at ~3 months. Patient 3 had no signs of remission or muscle-invasiveness before ~7 months following the first surgery. The patient was not operated with cystectomy for patient reasons and underwent palliative care for MIBC (**Fig 1D**). Immunofluorescence staining for PCNA and VEGF in the tumor samples of patients 3 and 8 confirmed the pathology report of disease progression (**S1 Fig**). As sample contamination from tumor proximity can be excluded for patients 1 and 8 (control sample collected ~7 cm away from the tumor site prior to tumor biopsy), these results suggest that seemingly normal tissue samples already hold clues of the disease progression towards muscle invasive tumors with extreme value for diagnosis and prediction.

### Proteomics confirms previously described molecular markers and signatures

We first validated our proteomic technique sensitivity by searching our assay for previously described molecular markers for bladder cancer [31–34] by quantifying the corresponding proteins to suggested genes (**S1 File**). We identified seven oncogenes (NRAS, EGFR, RXRA, HRAS, KRAS, AKT1, ERBB2; q<0.10) and seven putative tumor suppressor genes (ARID1A, TXNIP, CTNNB1, PIK3RI, CDKN2A, KLF5, STAG2; q<0.10) significantly regulated between tumor and control, which were formerly reported to be altered in BC [31]. Moreover, Chen *et al*. suggested seven candidate markers for bladder cancer, which were all identified by our assay (SLC3A2, STMN1, TAGLN2, Ca2, PGK1, SFN and TXN; q<0.10) [32]. These markers displayed significant regulations between tumor and control sample, despite not being aligned with the previously reported dataset on quantitative analyses of gene products.

Furthermore, Shimwell *et al*. suggested MDK and HAI-1 as markers of bladder cancer by transcriptome analyses of cell lines [34]. We quantified both gene products in our data, both significantly elevated in tumor relative to control (MDK T/C 13.55; HAI1 T/C 2.18, q<0.10).

Finally, TACSTD2 was described as a potent marker of NMIBC in urine [33]. Despite the different experimental setup, this gene product was up-regulated as expected from the urine assay (T/C 3.76, q<0.10).

Next, we performed KEGG pathway analyses of all quantified proteins [28–30] which showed a high coverage of genes annotated to be involved in the bladder cancer (**Fig 1E**), as well as associated pathways cell cycle, MAPK, TGF-beta, VEGF and p53 signaling (**S1 File**). Taken together, these results demonstrate that current global proteomics analyses can be used for the characterization of the bladder cancer fingerprint.

### Proteomics reveal a cancer-related molecular signature in the apparently healthy outlier samples, suggesting the subsequent MIBC onset

We then focused on the molecular fingerprint characterizing the two control outlier samples of patients 1 and 8 (N1 & N8, see **Fig 1A and 1B**) with the purpose of pinpointing candidates that could further be used for predicting cancer relapse and outcome.

**Fig 2:**
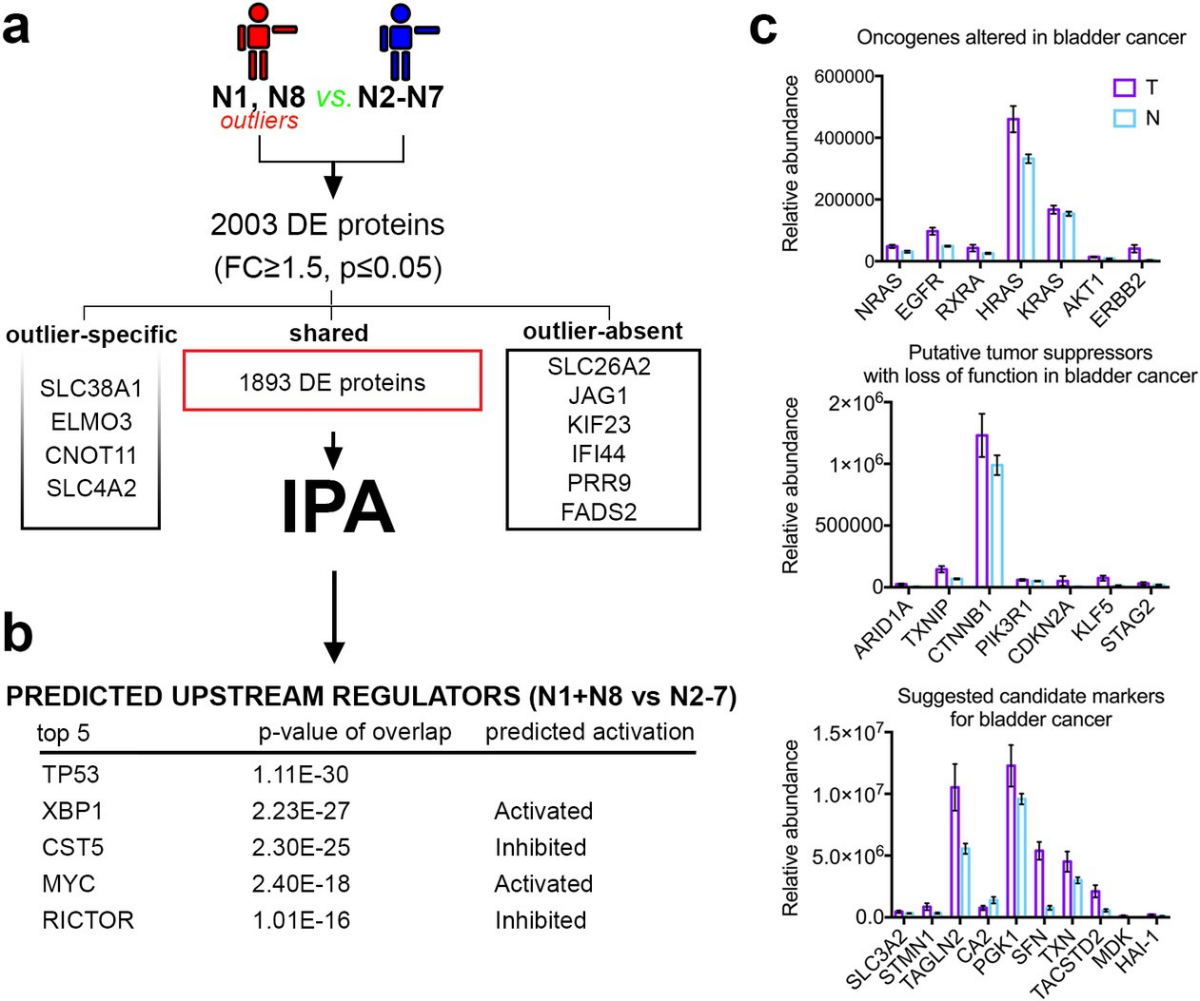
Global proteome analysis of the outliers (N1&N8) and control samples (N2 to N7). a) Scheme depicting the experimental design of the proteome comparison strategy. b) Table of upstream regulators predicted to be responsible for the deregulated proteomic fingerprint observed in the outlier samples. c) A graphical representation of previously described bladder cancer markers of tumor and control tissue with standard error of mean.

To achieve this, we first compared these apparently normal biopsies (N1 & N8) against the main cloud of control samples (N2 to N7) and detected 1903 differentially expressed proteins with four being expressed exclusively in the outliers, six exclusively in the normal cohort and 1893 being expressed in both (FC≥1.5, p≤0.05, **Fig 2A**).

In the outlier control samples, we identified proteins DNMT1 (DNA Methyltransferase 1), INSR (insulin receptor) and HDAC1 (histone deacetylase 1) displaying a regulation pattern previously associated with different cancer types, including MIBC. DNMT1, the major methyltransferase responsible for DNA methylation, displays a 6.2-fold upregulation (q = 0,019) in the outliers (N1 & N8) as compared to the normal N2 to N7 cohort. DNMT1 is overexpressed in many types of cancer [42–44] including MIBC [45, 46], and it is also a target for chemotherapy [47]. INSR, upregulated 3.15-fold (q = 0,027) in the outliers, was reported to be overexpressed in several cancer types and was associated with poor prognosis when overexpressed on tumor-associated blood vessels in MIBC [47]. HDAC1 (FC = 1.63-fold, q = 0,082) is overexpressed in many cancers such as colon and testis and its overexpression was associated with high-grade tumors and poor prognosis in bladder cancer [48].

This observed up-regulation of proteins with known overexpression in cancers in apparently healthy tissue samples suggests that subtle but critical molecular changes are initiated in the bladder long before the tumor growth. These changes could be used as markers for disease prognosis.

To further extend and explore this molecular signature in an unbiased fashion we performed pathway analysis of the differentially expressed protein list by using the Ingenuity Pathway Analysis software (**Fig 2A**). This predicted Cancer and Tumor morphology between the top 5 disease and disorders. Notably, TP53 was predicted as top upstream regulator, followed by XBP1, CST5, MYC, and RICTOR (**Fig 2B**). XBP1 and MYC, both known promoters of tumorigenicity and cancer progression in diverse cancer forms [49, 50], were predicted as activated. Moreover, CST5, a direct target of TP53 contributing to its tumor suppressor role, was predicted as inactivated, consistent with a cancer promotion regulation [51]. The presence of several key tumor modulators among the top 5 upstream regulators, predicted to be responsible for the proteomic fingerprint observed in the outlier samples, suggests an early shift of this apparently normal tissue towards a cancer phenotype and it is consistent with the PC plot grouping towards the cancer biopsies (**Fig 1B**). A graphical representation of protein expression data from previously described bladder cancer markers are shown in (**Fig 2C**)

### Half of the outliers’ deregulated proteins display similar expression levels in BC samples

**Fig 3:**
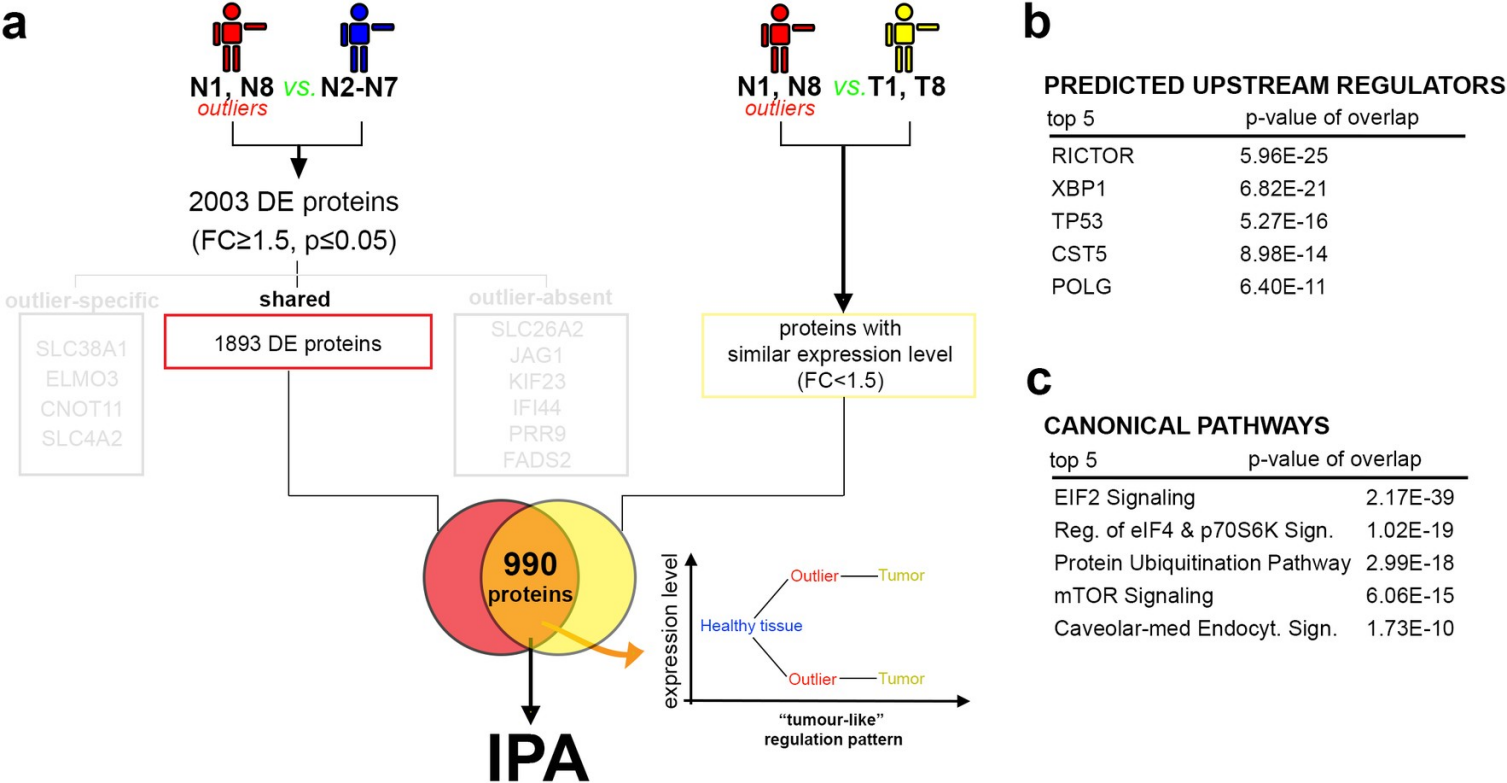
Global proteome analysis of similarities between the outlier and tumor samples. a) Scheme depicting the experimental design of the proteome comparison strategy. b) Table of the predicted upstream regulators responsible for the deregulated proteomic fingerprint observed in the outlier samples. c) Table of the canonical pathways revealed by the pathway analysis in the analyzed DE protein list.

To establish how similar the identified molecular signature is to a typical tumor fingerprint, our protein signature was further compared to the proteome landscape characterizing their respective tumor samples (T1 & T8). We first focused on proteins that by their deregulation in the outliers (N1 & N8) reached similar expression levels with the ones measured in the bona fide tumors (**Fig 3A**). 990 proteins (~53%,) followed this “tumor-like” pattern of regulation (i.e., reached an expression level similar to the one detected in the tumor samples), including HDAC1 and INSR. The Ingenuity Pathway Analysis (IPA) of this subset predicted cancer as top disease and disorder, RICTOR, XBP1, TP53, CST5 and POLG as top upstream regulators (**Fig 3B**) and the upregulation of mTOR pathway in the top 5 canonical signaling pathways (**Fig 3C**). These data confirm the outlier status of the N1 and N8 control samples, while explaining their clustering closer to the tumor samples. However, as just about half of the deregulated proteins in the outliers reached expression levels similar to the ones recorded in the tumors, these samples also present their own distinct proteome signature.

### Several outliers-specific deregulated proteins show overexpression compared to tumor samples

**Fig 4:**
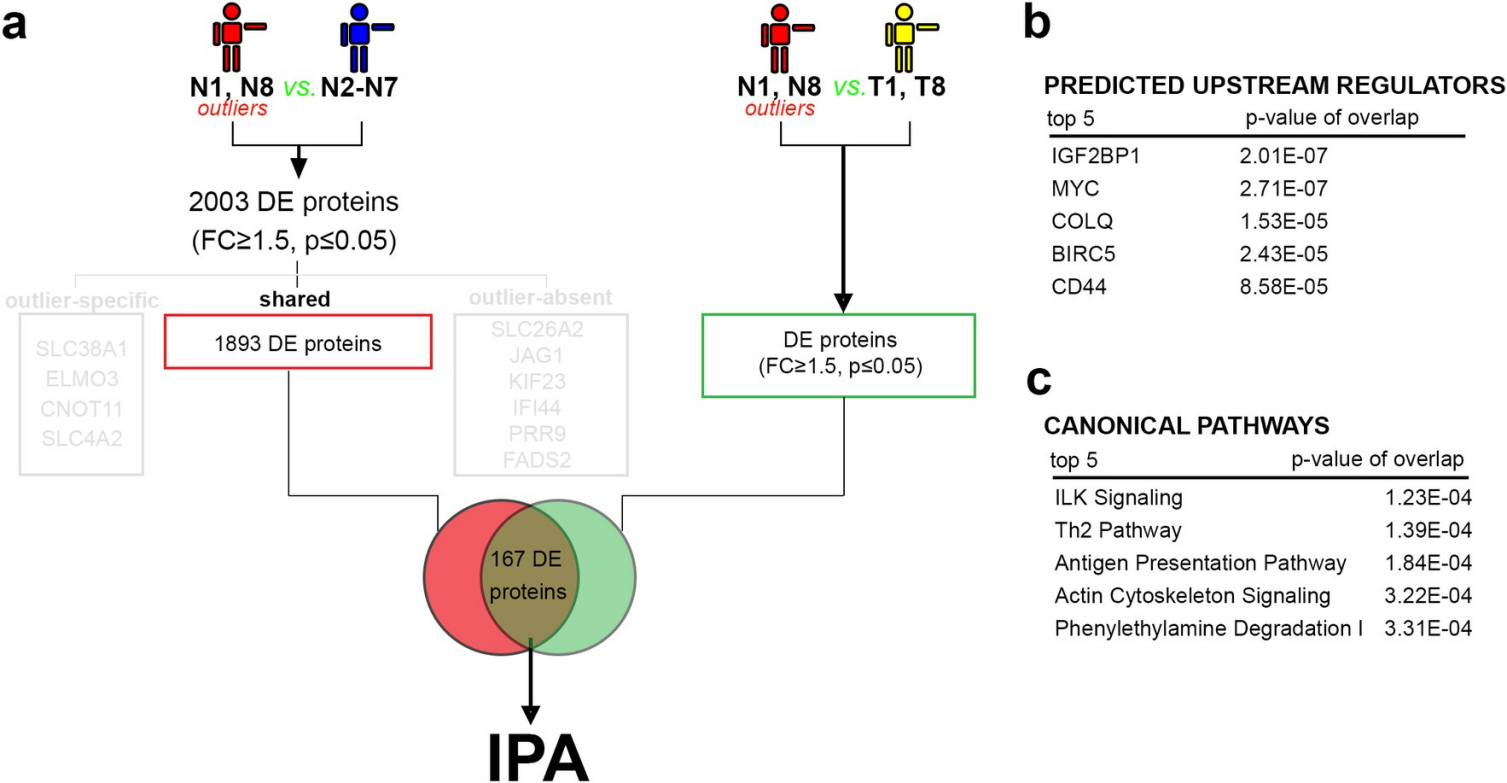
Global proteome analysis of “outlier-specific” molecular signature. a) Experimental design of the proteome comparison strategy. b) Table of the predicted upstream regulators. c) Table of the canonical pathways.

In order to characterize the “outlier-specific” molecular signature and as such identify specific markers allowing the identifications of bladder cancers with similar evolution, we further focused on proteins that by their deregulation in N1 and N8 presented different expression patterns in the tumor (**Fig 4A**). We identified 167 (9%) proteins (FC≥1.5 p≤0.05) overexpressed in outliers as compared to both the normal control samples (N2 to N7) and tumor samples (T1 & T8).

DNMT1 was identified in this protein group, showing overexpression both compared to the healthy control samples (FC = 6.20x, see above) and NMIBC samples (FC = 3.22). Daintain/AIF1 (allograft inflammatory factor) is a crucial mediator hub of inflammatory response and cell migration [52], and an overexpression of this protein has been reported to be involved in several cancers [53–55]. AIF1 was overexpressed in the outlier samples as compared to both normal control samples (FC = 6.89x q = 0,053) and tumor samples (FC = 3.29x). Similarly, ICAM1 (Intercellular adhesion molecule) is also associated with cancer progression and invasion, and was overexpressed in the outliers (6.64x higher than in control samples (q = 0,00025) and 3.12x higher in tumor samples).

The IPA analysis strength was limited due to the low number of proteins in this profile, however the disease and biological function analysis predicted the involvement of the analyzed proteins in the activation of cell signaling, cell motility, extracellular matrix and immune cell trafficking. Moreover, IGF2BP1, MYC, COLQ, BIRC5 and CD44 were predicted in the top 5 upstream regulators (**Fig 4B**). The ILK signaling pathway was identified as the top canonical pathway of this profile, being predicted as activated (**Fig 4C**). The outlier-specific set’s proteins suggested involvement in inflammation, cell-to-cell communication and motility, which could explain the fast development of the muscular invasive form of cancer in the analyzed patients 1 and 8, with remission at 3 months.

## Discussion

This pilot study suggest that macroscopically normal tissue is not necessarily molecularly “healthy”. This has important implications for diagnosis concerning the importance of collecting the apparently healthy tissue of a cancer-invaded organ for its potential value in predicting the evolution of the disease. Moreover, analyzing cancer markers in an otherwise macroscopically free cancer environment offers the possibility to analyze subtle molecular changes, which will be otherwise covered by the tumor mass signature.

There is a current lack of adequate markers in regard to predicting the switch from the NMBIC to the metastasis prone MIBC. Using a global proteomic approach, we analyzed control healthy tissue and tumor tissue form 8 primary NMBIC patients. We observed that for two of them (patient 1 and 8), the two apparently normal control samples, behaved differently as compared to the other control samples, clustering closer to the tumor samples cohort. Interestingly, these two patients relapsed very soon following the intervention, this time with MIBC. By performing comparison analyses of presumed normal tissue from these outlier samples we were able to detect specific protein markers overexpressed as compared to typical healthy tissue. Moreover, some of these markers had higher expression in the control tissue than in the NIMBC tumor sample. We detected proteins DNMT1, ICAM1 and AIF1 as being highly expressed in the outlier presumed normal tissue compared to both other controls and tumor tissue from the same patients.

The high incidence of bladder cancer as well as the subsequent need for life-long surveillance exercises a strong pressure for finding early diagnosis and prognosis tools, which will allow for a better management of this disease. Establishment of molecular signatures for risk stratification would reduce mortality as well as morbidity due to both disease and treatment, and allow for better use of healthcare resources by the application of personalized therapy treatment algorithms. Certainly, further studies on larger cohorts of patients are required to confirm the value of macroscopically cancer free samples in predicting cancer prognosis, and the observed association between subtle changes in their molecular pattern and tumor evolution.

This study presents obvious, however unavoidable, limitations. Due to the rather low number of patients (n = 8) and the heterogeneous nature of the disease, the results will need further validation in large-scale studies. For such a small study, the p-value will overestimate number of real significance, while the q-value will underestimate. It is worthwhile to mention the use p-values were not used as a measure of statistical significance, but rather as a cutoff to identify the most interesting proteins (Figs 2A, 3A and 4A). Moreover, this pilot highlighted new requirements for the design of future studies, such as the necessity of *in situ* validation (histology or immunofluorescence) on the exact same specimen used for the omics assay. This corroboration was impossible using the current basic design. This is in particular notable in this manuscript given the inability to fully explain the nature of each tumor. Observing Fig 1B, a particular peculiarity is the grouping of sample T3 in the PC-plot that may or may not be a result of sample heterogeneity. Finally, the layout using a sole biopsy of normal and control tissue from each patient is prone to bias due to both the heterogeneity of bladder cancer and bladder cancers as a field disease.

## Supporting information

S1 File Supplementary material. The supplementary material includes i) An overview of the chromatography settings and instruments for the LC-MS/MS experiments ii) Our search results from previously suggested markers (ref 31–34) and iii) A summary of data searches from KEGG pathway analyses (ref 28–30) which showed a high coverage of genes annotated to be involved in the bladder cancer, as well as associated pathways cell cycle, MAPK, TGF-beta, VEGF and p53 signaling.
(DOCX)

S1 Fig Supplementary figure. Immunofluorescence staining for PCNA and VEGF in the tumor samples of patients 3 and 8 confirmed the pathology report of disease progression.
(TIF)
