# Supplementary material for: Novel protein signatures suggest progression to muscular invasiveness in bladder cancer
Source: PLoS One. 2018 Nov 12;13(11):e0206475. doi: 10.1371/journal.pone.0206475 (PMC6231613; doi:10.1371/journal.pone.0206475)

**i) Settings used for chromatography**

Chromatography settings:

| Mass spectrometre | Q-Exactive HF |
| --- | --- |
| LC system | Dionex Ultimate NCR-3500RS |
| Ion source | EASY-spray |
| Instrument control | Q-Excative HF Tune 2.4 and Xcalibur 3.0 |
| Pre-column | Acclaim PepMap 100, 2 cm x 75 µm i.d, 3 µm C18 beads |
| Column | PepMap RSLC, 25cm x 75 µm i.d. EASY-spray column, packed with 2µm C18 beads |
| Solvent A | 0.1% FA (vol/vol) in water |
| Solvent B | 100% ACN |

Chromatography gradient

| **%B** | **Time** | **Remarks** |
| --- | --- | --- |
| 5% | 5min | Trapping |
| 5-8% | 0.5min |  |
| 8-24% | 109.5min |  |
| 24-35% | 25min |  |
| 35-90% | 15min |  |
| 90% | 15min | Hydrophobic + conditioning |
| 5% | 20min | Column conditioning |

**ii) Our search results from previously suggested markers (ref 19-22)**
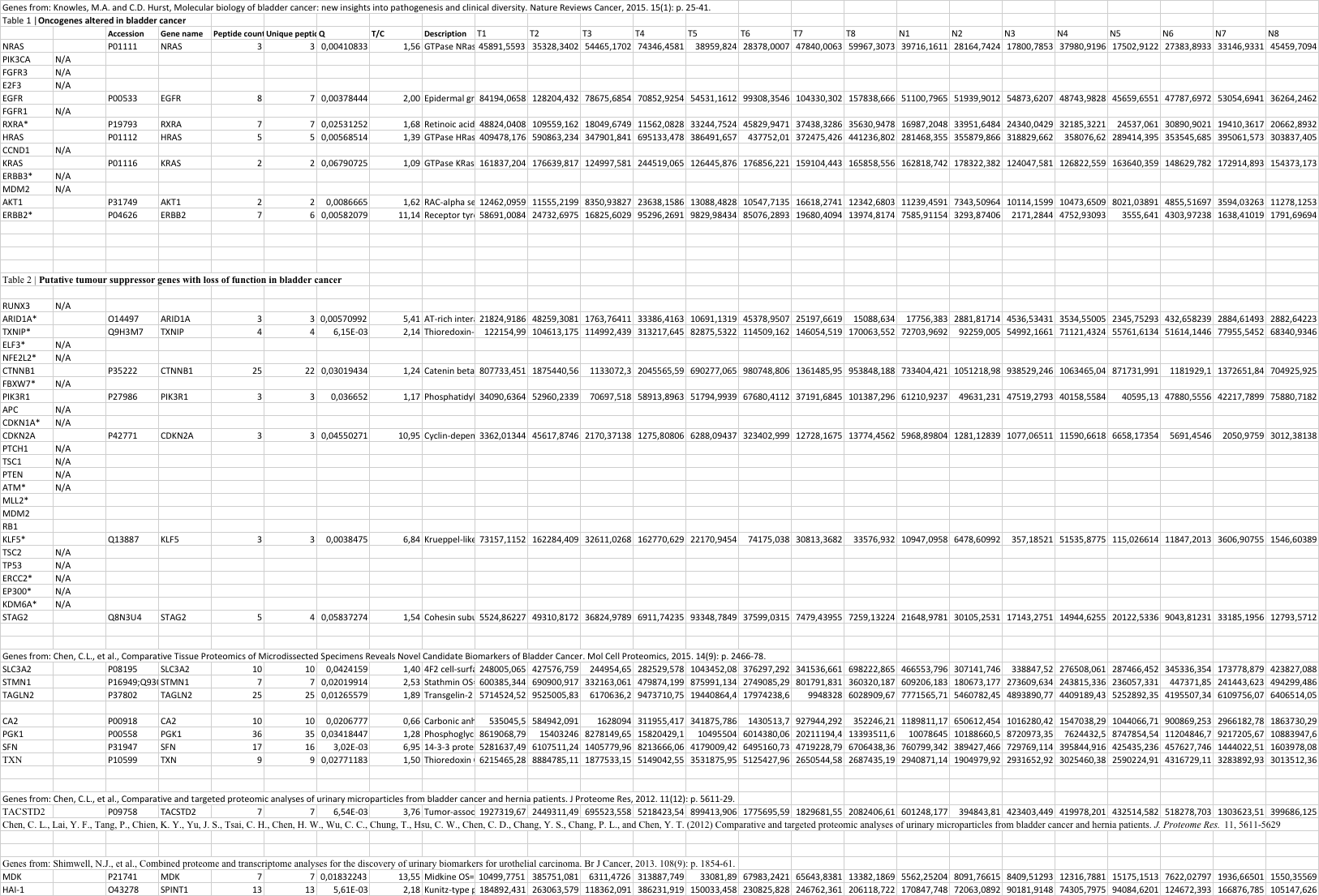


**iii)** **KEGG pathway analyses (ref 23-25) which showed a high coverage of genes annotated to be involved in the bladder cancer, as well as associated pathways cell cycle, MAPK, TGF-beta, VEGF and p53 signaling**


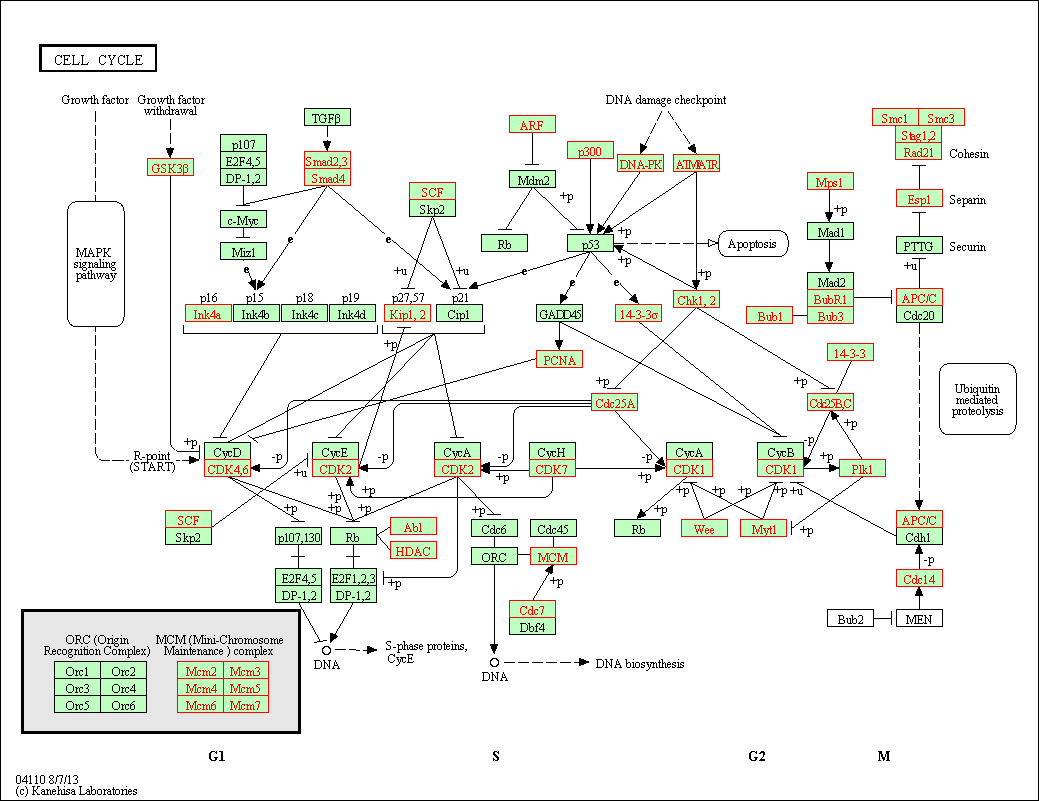

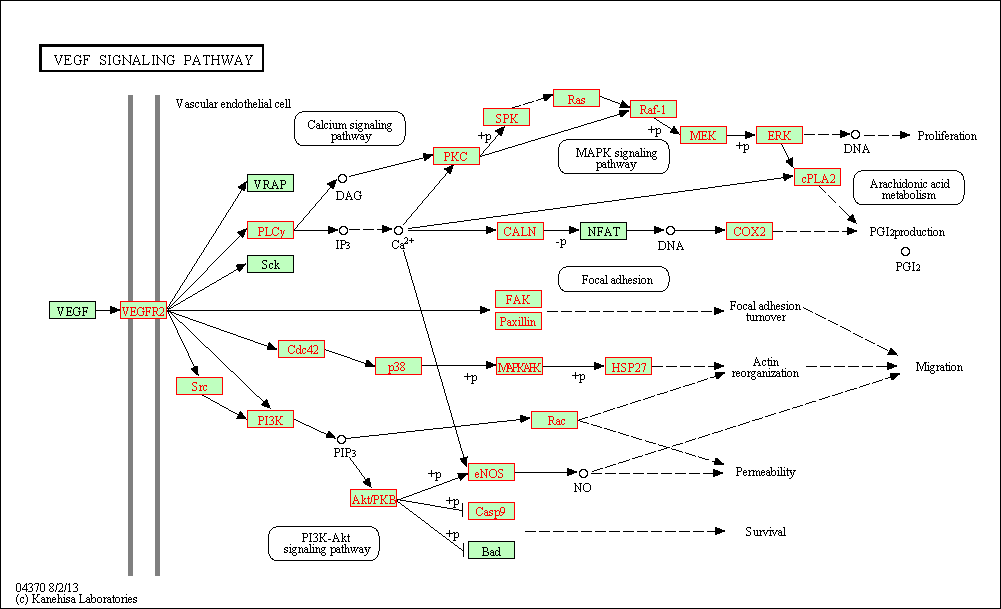

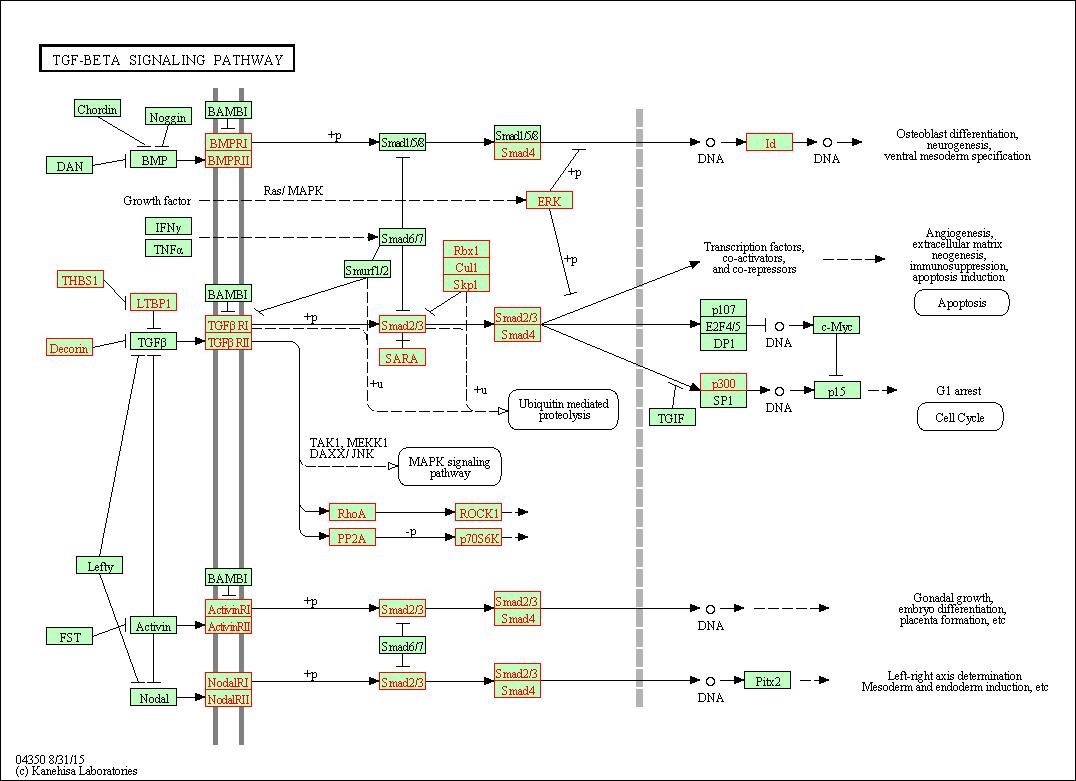

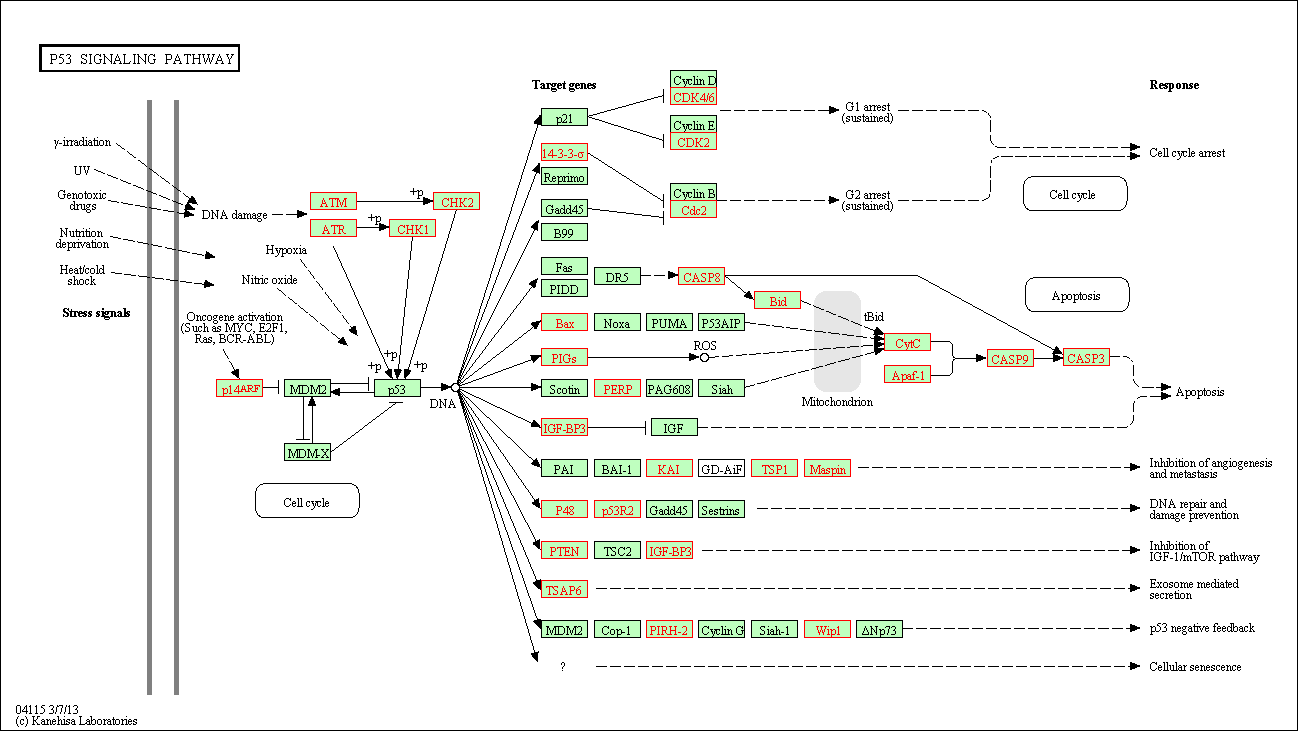

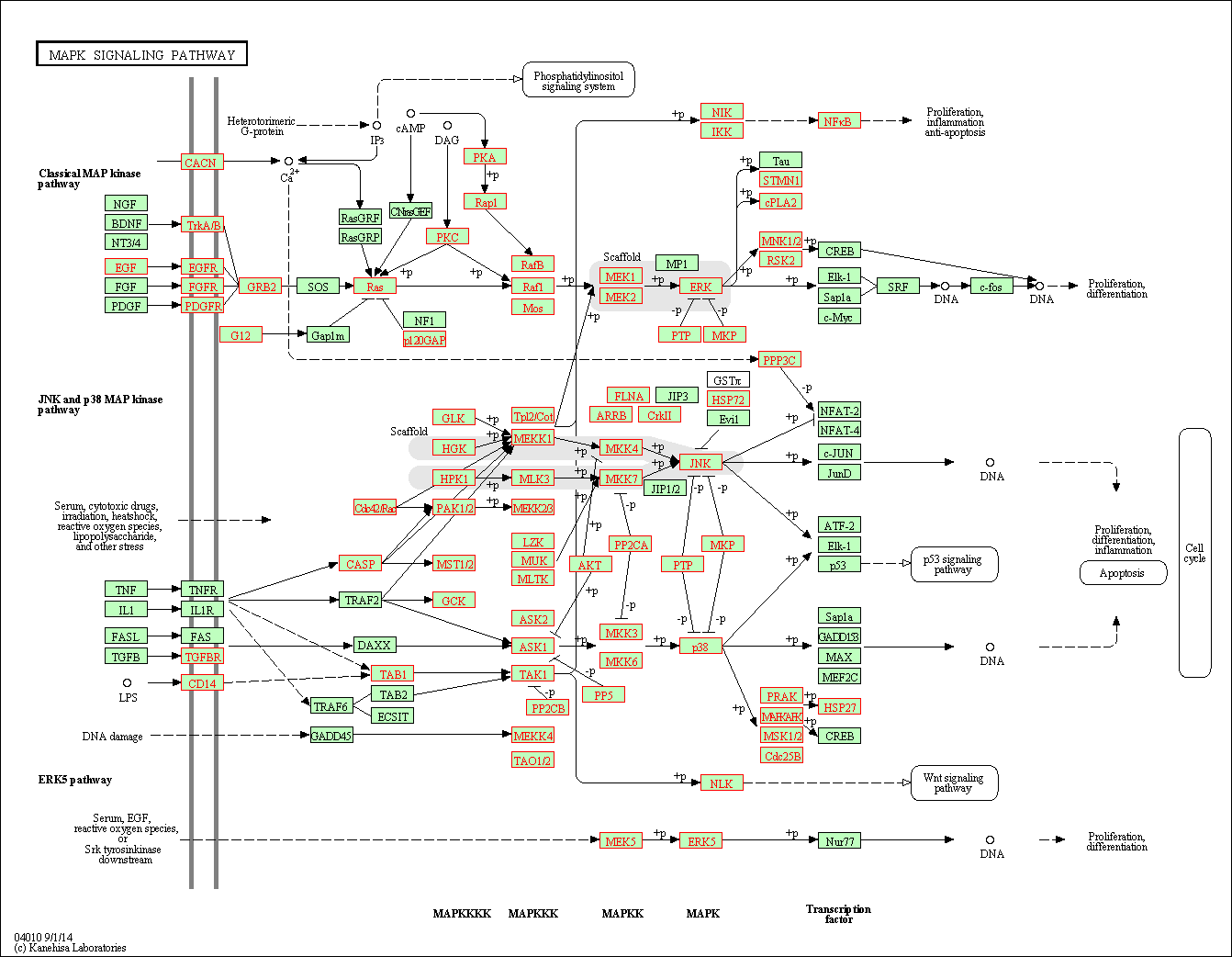

Supplement: S1 File — The supplementary material includes i) An overview of the chromatography settings and instruments for the LC-MS/MS experiments ii) Our search results from previously suggested markers (ref 31–34) and iii) A summary of data searches from KEGG pathway analyses (ref 28–30) which showed a high coverage of genes annotated to be involved in the bladder cancer, as well as associated pathways cell cycle, MAPK, TGF-beta, VEGF and p53 signaling. (DOCX) [file pone.0206475.s001.docx]
