# Supplementary figures and images for: Novel protein signatures suggest progression to muscular invasiveness in bladder cancer
Source: PLoS One. 2018 Nov 12;13(11):e0206475. doi: 10.1371/journal.pone.0206475 (PMC6231613; doi:10.1371/journal.pone.0206475)

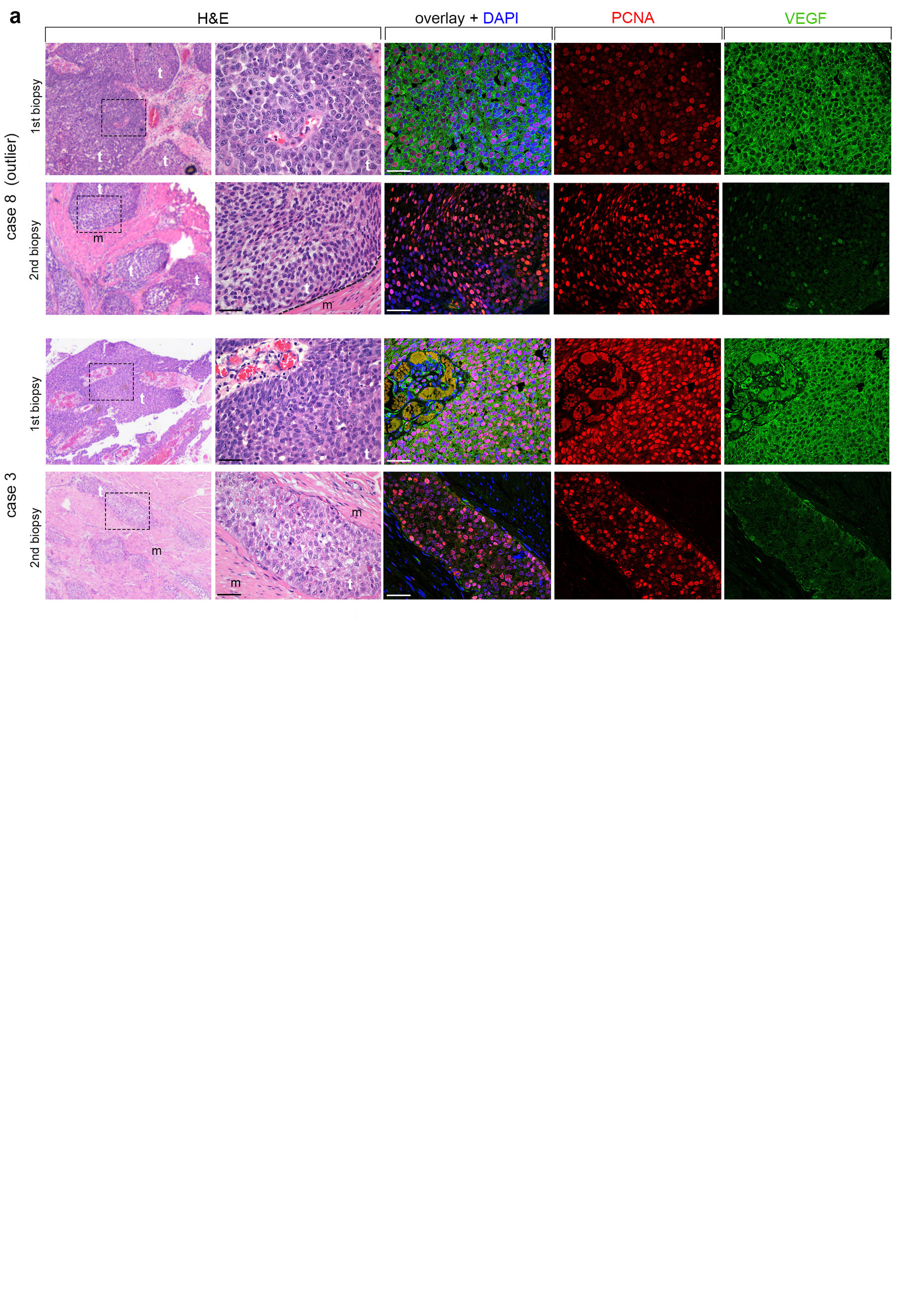

Supplement: S1 Fig — Immunofluorescence staining for PCNA and VEGF in the tumor samples of patients 3 and 8 confirmed the pathology report of disease progression. (TIF) [file pone.0206475.s002.tif]
